# ANOVA and design expert model for discoloring of azo benzene derivative dye used mesoporous aluminum silicon oxide nanoparticles

**DOI:** 10.1038/s41598-025-27886-x

**Published:** 2025-12-20

**Authors:** Yara M. Adly, Magdi E. A. Zaki, Omar A. Fouad, Gehad G. Mohamed, Sami A. Al-Hussain, Maysa R. Mostafa

**Affiliations:** 1 Chemistry Department, Faculty of Science, Cairo University, Giza, 12613 Egypt; 2 Nanoscience Department, Basic and Applied Sciences Institute, Egypt-Japan University of Science and Technology, New Borg El Arab, Alexandria, 21934 Egypt; 3 Department of Chemistry, Faculty of Science, Imam Mohammad Ibn Saud Islamic University (IMSIU), 11623 Riyadh, Saudi Arabia

**Keywords:** Nano mullite, Methyl red, Adsorption, Kinetics, Isotherm, ANOVA-design expert, Environmental sciences, Chemistry, Materials science, Mathematics and computing

## Abstract

Modeling using statistical methods as ANOVA and design expert help in predicting, improving, and perfecting a system’s behavior by integration between all experimental data and different parameters, in addition to it doesn’t consume time or materials as the other techniques statistically independence. It investigates the adsorption effectiveness of mullite nanoparticles (MNPs) in removing methyl red dye (acid red 2) as azo benzene derivative dye. Many parameters as pH, dose, contact duration, and dye concentration were studied. The crystal structure, morphology, nanoscale and surface area of the adsorbent MNPs material were investigated using X-ray diffraction, Brunauer-Emmett-Teller, and transmission electron microscopy methods. The adsorbent had high crystallinity with particles size of 5 to 25 nm and an average equal 12.3 nm. In addition, it had mesoporous characteristics with high surface area (an average pore size of 7.224 nm, 93.71 m^2^/g of surface area, and 0.426 cm^3^/g of pore volume). Its contact angle is 115.3, which explains its hydrophobic character. By using a dosage of 0.05 g at pH 4, for 10 min and 800 rpm, more than 99% removal was achieved. Application of the adsorption isotherm and Kinetics, it follows pseudo-2nd order and the DKR isotherm of the removal procedure.

**Supplementary Information:**

The online version contains supplementary material available at 10.1038/s41598-025-27886-x.

## Introduction

**Fig. 1:**
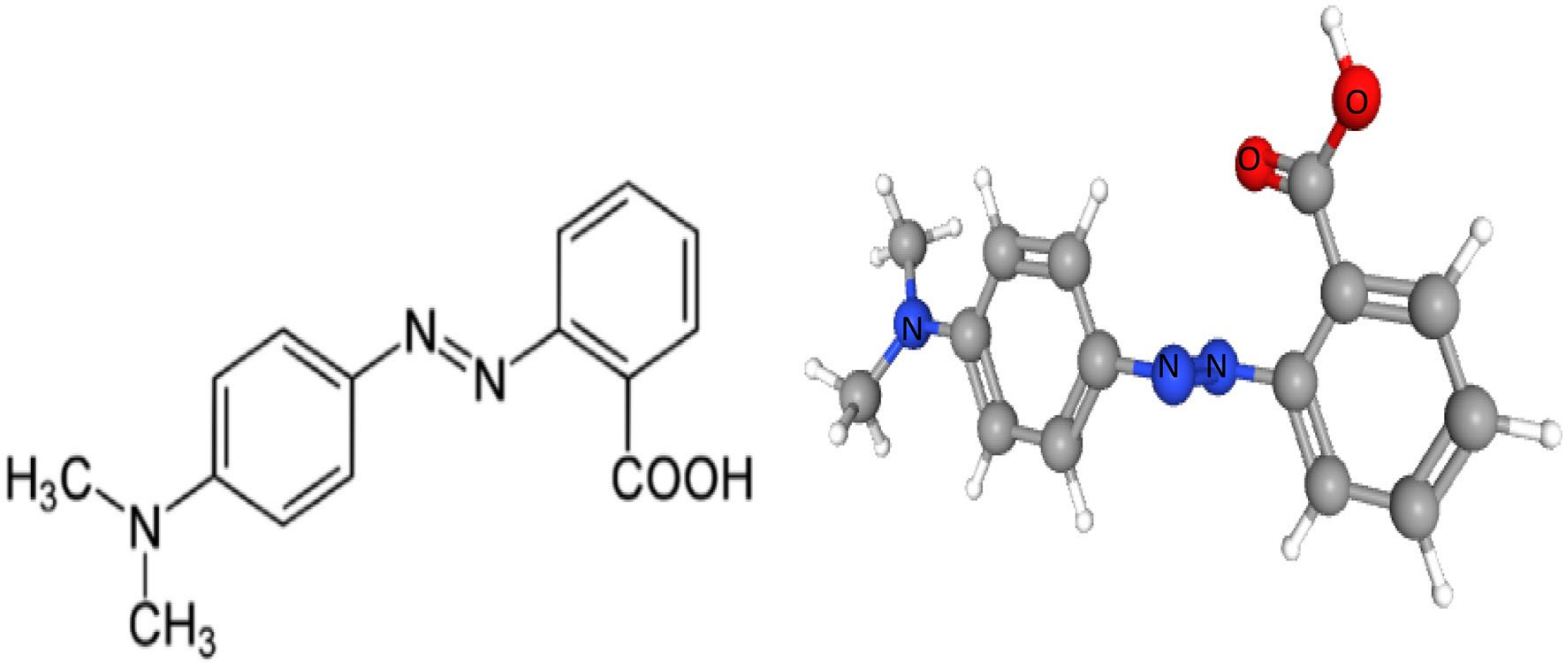
1D, 3D Structure of methyl red (ACID RED 2)^4^.

In many different industries, including textiles, printing, plastics, cosmetics, and paper, dyes are widely used. Two classes of chemicals include (i) chromophores as colorants and (ii) auxochromes for intensity^1,2^. The textile industry is the one that produces the most dye wastewater that enters aquatic systems between these industries^3^. The release of hazardous compounds by dye compounds can harm individuals and aquatic ecosystems, making dye industrial wastewater a serious issue. It is estimated, then, that more than 700,000 tones are produced annually all around and that more than 10,000 distinct dye kinds are employed in business^1,4,5^. Between these dyes, methyl red (MR) dye or acid red is a monoazo dye as shown in Fig. 1 which explain the 2D and 3D structure of the acid red 2 dye^6^, and one of the dyes that is most frequently used in laboratories as an acid-base indicator^7^. Anionic azo dyes like methyl red pose significant environmental challenges due to their toxicity and persistence. MR is an anionic azo, which is very poisonous to aquatic life and has long-term consequences. Its persistence in the environment may potentially have negative repercussions for human health. It is considered toxic if eaten and may cause skin sensitization. Furthermore, it is considered a possible carcinogen, with some research indicating that it may be mutagenic and induce chromosomal abnormalities. Its breakdown products, especially aromatic amines, can be hazardous^8,9^.

Various methods have been employed thus far to handle wastewater^10,11^ as membrane filtration^12^; microbiological degradation^13^; adsorption^14,15^; coagulation^16^; photocatalytic and electrocatalytic degradation^17,18^ and precipitation^19^.

Although sophisticated oxidation techniques and biological processes are great chemical-free solutions, they have limitations in terms of scalability and by-products^20^. The use of chemicals, the production of secondary pollutants, and the inadequate removal of dyes are some disadvantages of chemical precipitation, despite its low operating costs. Photocatalytic degradation also effectively removes the pollutants with minimal chemical consumption; nevertheless, it has limitations, such as limited light penetration, slower reaction kinetics, and the production of byproducts^21,22^. On the other hand, because of its ease of use, environmentally benign methodology, and effective removal of organic dyes and inorganic contaminants, the adsorption process is commonly regarded as the most efficient and cost-effective way for treating water^3,23^. It can achieve wastewater purification as well as the recycling of organic dyes. Furthermore, low expenses, simple operations, absence of hazardous materials, and extensive process efficiency also satisfies the criteria set forth by green chemistry^23,24^.

Adsorption behavior can happen in several stages, including the movement from bulk to the surface of adsorbent. Pore, surface, or an integration of many processes, external diffusion, and pore surface adsorption can help to control it^3^. Porous ceramics^25–27^, such as mullite, have found use in industry as filters, catalysts, and light structural supports.

Nanoparticles have shown promise as adsorbents due to their high surface area, thermal stability, and surface charge characteristics which may be used in many applications as determination of substances or removal process^28–31^. Mullite nanoparticles (3Al₂O₃·2SiO₂) have shown promise as adsorbents due to thermal stability, high surface area, and surface charge characteristics. The numerous extraordinary qualities of mullite are high temperature strength, low thermal expansion coefficient, oxidation-resistant, high heat resistance, and more. As support for ceramic matrix composites, it works rather well^32^. Mullite ceramics (MNPs) possess many useful uses in several sectors, including crucibles, heat exchangers, dental ceramics, hot gas filters, electronic packaging, and silicon solar cell substrates^33^. The specific temperature at which mullitization occurs is directly affected by the mullite synthesis process, which determines the exact ratio of mixing for silicon and aluminum in the precursor^34^. A variety of techniques, including gel-casting^35^, freeze-casting^36^, direct-foaming^37^, replica^38^, starch consolidation^39^, thermal gelation^40^, foam-casting^41^, and sol-gel method^25^, were employed to prepare mullite nanoparticles. These ceramic nanoparticles were created via sol-gel method^28^. This study used sol-gel procedures to synthesis the nano mullite, which was subsequently calcined at 1000 °C and evaluated using Brunauer–Emmett–Teller (BET), transmission electron microscope (TEM) and X-ray Diffraction (XRD) analyses. Utilizing design expert and ANOVA software to optimize the factors was performed. An adsorbent made of MNPs to eliminate acid red 2 dye was used. The purpose of this work is to characterize and optimize wastewater dye adsorption on nano mullite. Among the objectives are figuring out the thermodynamics, reusability, isotherms, and adsorption kinetics.the final dye concentration was determined spectrophotometrically to match the dye λ_max_ of 515 nm using the Beer- Lambert equation^42^.

This study employs statistical design of experiments to model and enhance the adsorption process of mullite nanoparticles (MNPs). Solving equations with several variables is achievable through the integration of experimental data and statistical design methodologies. Optimizing all process parameters collectively aids in forecasting, enhancing, and refining a system’s performance. It transcends the constraints of traditional experimental techniques and assesses the cumulative impact of all variables.

To reduce material usage and processing duration, ANOVA and Design Expert used as statistical method that predict the best model including the most effective parameters that have a critical influence on the performance of the adsorption mechanism. The validation using ANOVA (Analysis of Variance) provide a complete validation report about many statistical parameters as F-, p- values, R^2^, adjusted R^2^, standard error, confidence interval and residuals. Besides, it expresses the interaction between the factors and informs which parameters have a great effect. In addition to give a complete statistical analysis for the results with different graphical plots as normal probability, cox-box, ramp plot, 3D curves….etc which enable to achieve the maximum removal capacity with lowest amount of adsorbent within short time that progress the green and sustainable method^43–45^.

This study introduces mullite nanoparticles as a unique and effective adsorbent for the removal of methyl red dye, utilizing statistical optimization (ANOVA and Design Expert) integrated method exhibits exceptional removal effectiveness (>99% within 10 min at minimal dosage) and offers a scalable and sustainable solution for azo dye wastewater treatment.

## Experimental data

### Materials and instruments

Supplementary data covers all of the substances, reagents, and equipment used.

### Preparation of mullite nano-ceramic

**Fig. 2:**
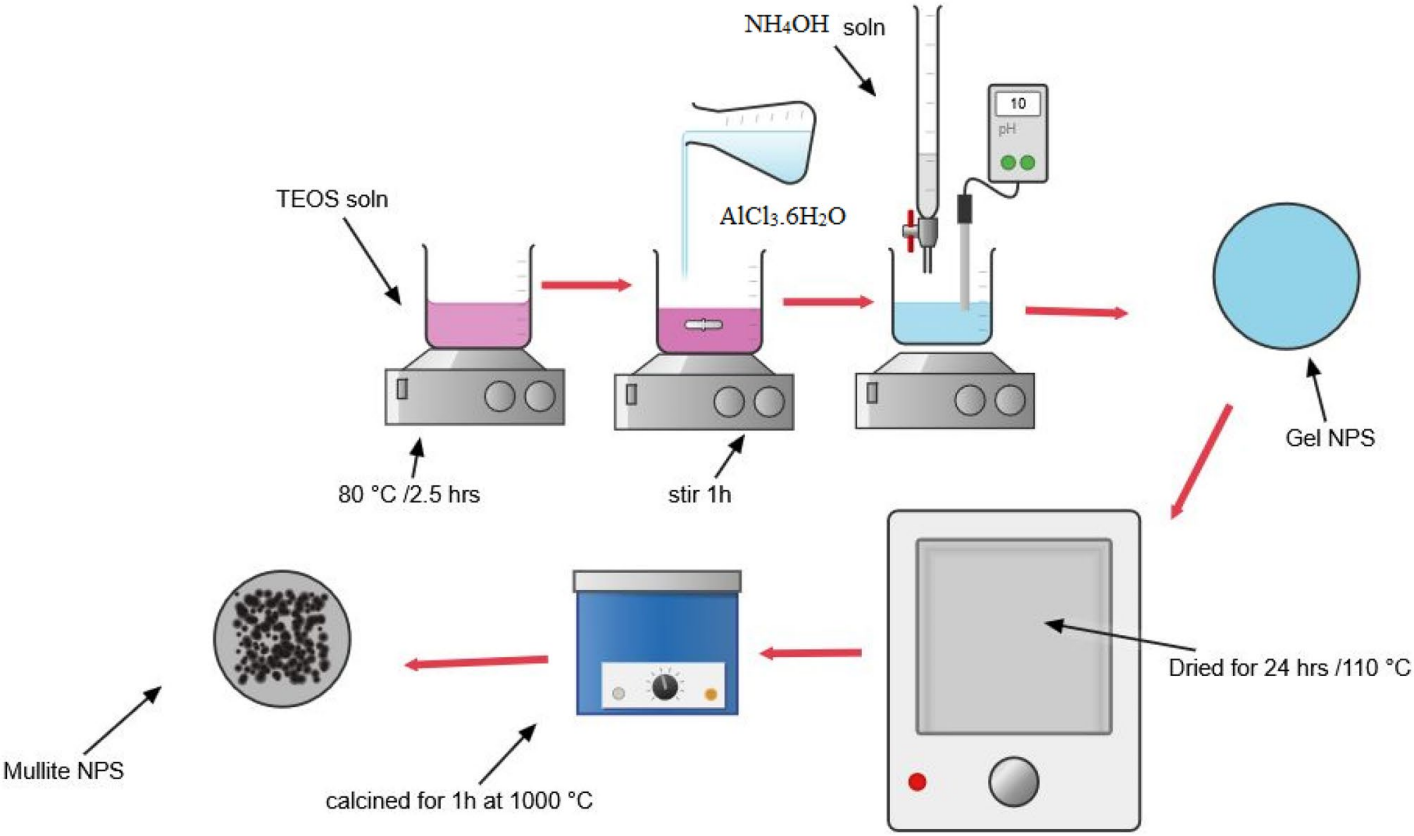
The synthesis method of the nanomullite.

The sol-gel method was used to synthesize these ceramic nanoparticles^25^. As shown in Fig. 2, the synthesized method was done by 1M concentration solution of aluminum chloride hexahydrate (AlCl_3_·6H_2_O) was prepared by continuous stirring of water. To achieve complete hydrolysis of silica, a mixture of TEOS solution, water, and ethanol was prepared in a 1:4 molar ratio and stirred at 80 °C for 2.5 hrs. The homogeneous solution was subsequently mixed for one hour with an aluminum chloride hexahydrate (AlCl_3_·6H_2_O) solution at a molar ratio of 3:2 for Al_2_O_3_ to SiO_2_. The pH of the combined solution was maintained at 10 through the gradual addition of ammonium hydroxide solution. Subsequently, the gel was formed at room temperature, dried for 24 hrs at 110 °C in an oven, and then calcined for 1 h at 1000 °C^46^.

### Research concerning batch adsorption

By utilizing the batch adsorption approach, the dye was successfully removed from the adsorbent. The batch adsorption experiment used 100 mL of dye solutions and 0.05 g of adsorbent at a constant temperature of (25±1) °C. 0.1 mol.L^−1^ HCl and 0.1 mol.L^−1^ NaOH solutions were added to the tested solution to change its pH. The mixed solution was shaken continuously during the studies. After the necessary amount of time, the adsorbent was removed from the solution by filtering through a 45µm polyethylene membrane filter. The length of contact, starting dye concentration, agitation speed, adsorbent dose, solution temperature, and pH level were among the several factors that were examined.

- A Using the following formula, we were able to determine the dye clearance percentage:

1 $$\% Removal = \, (C_{0} - C_{e} ) \, /C_{0} \times { 1}00$$

Where C_e_ is the dye concentration resulting from adsorption (ppm), and C_0_ is the dye concentration at the beginning (ppm)^47^.

-The capacity of adsorption, q_e_ (mg d per g dry adsorbent), using the following equation:

2 $${\text{q}}_{{{\text{max}}}} = \, \left( {{\text{C}}_{0} {-}{\text{C}}_{{\text{e}}} } \right) \, \times \left( {{\text{v}}/{\text{w}}} \right)$$

since, W is the dry adsorbent’s weight in grams, and V is the solution’s volume in liters^47^.

### Statistical analyses

The parameters for dye elimination were modeled and optimized using ANOVA and design expert programs^48^. In order to foretell how responses would play out after chemicals have been removed, the investigations depend on realistic simulations. Based on these programs, the most successful option is the adsorption-based removal approach (RDA), which can produce a prototype with little experience necessary. The time and money spent on experiments are also reduced.

## Discussions and results

### XRD

**Fig. 3:**
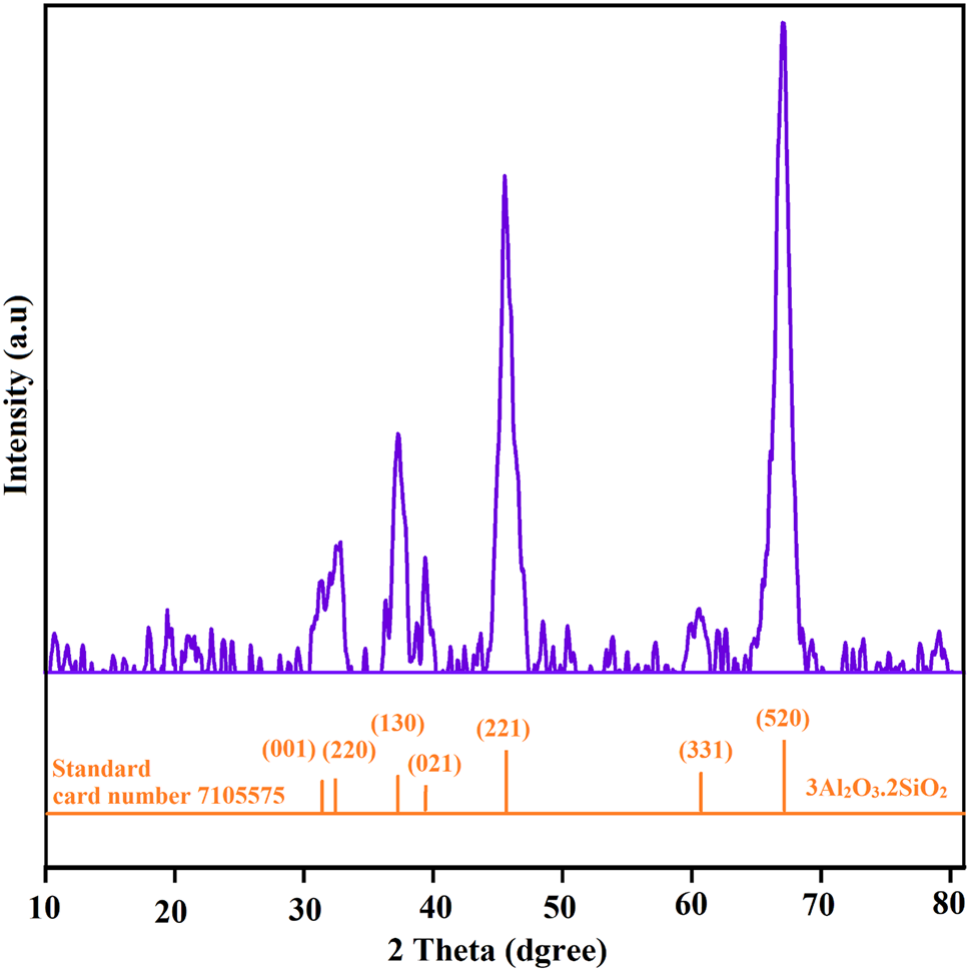
Nano mullite X-ray diffraction patterns.

XRD verified the existence of nano-mullite (3Al_2_O_3_.2SiO_2_) as shown in Fig. 3. The most intensity peaks of the XRD pattern designated by card number 7105575, crystals shape is orthorhombic in the P b a m (55) space group of the nano-mullite (3Al_2_O_3_.2SiO_2_)^25^. These diffraction peaks appear at 31.06°, 33.07°, 36.84°, 39.04°, 46.23°, 60.24°, and 66.20°represented by planes (0 0 1), (2 2 0), (1 3 0), (0 2 1), (2 2 1), (3 3 1), and (5 2 0) respectively. The nano size of the prepared nano-mullite (3Al_2_O_3_.2SiO_2_) was confirmed by applying Debye-Scherer equation in the XRD pattern (Supplementary Table S1) and was found that the crystallites was varied in size from 6.08 to 12.01 nm, with an average of 12.34 nm^25,46,49,50^.

### TEM

**Fig. 4:**
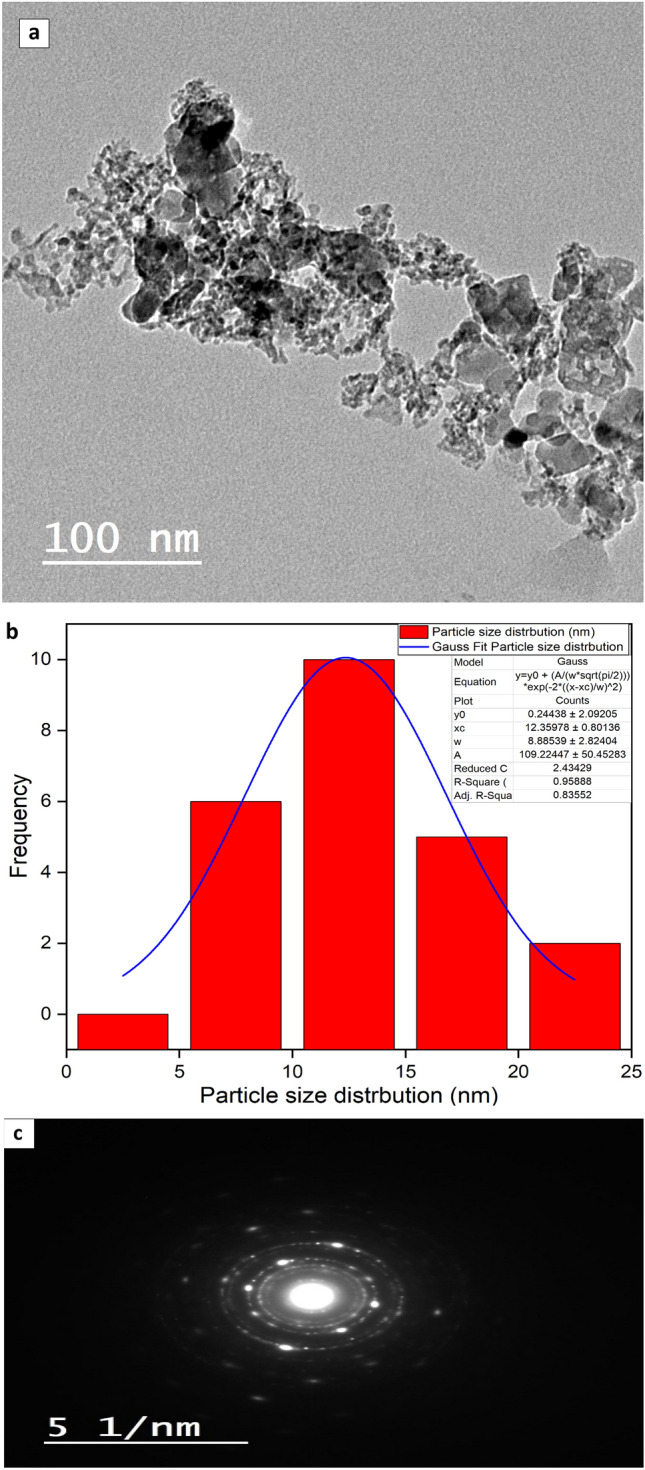
(a, b and c). (**a**) (HR-TEM) with distribution of particle size (**b**), and (**c**) SAED for MNPs.

To examine surface features and particle sizes for the produced MNPs, the analysis as transmission electron microscope was performed. The high-resolution TEM image (HR-TEM) for the mullite nanoparticles’ confirm the crystalline characteristics of prepared mullite nanoparticles as shown Fig. 4a also the selected area electron diffraction (SAED) of nano mullite as shown in Fig. 4c, clearly shows a highly crystalline level as seen by the material’s well-organized lattice edge arrangement. A Gaussian mixture model and histogram created using the Java 1.8.0 172 with ImageJ (1.53e) tool also reveal their particle size as in Fig. 4b^25,26,51^. The size of the particles was from 5 to 25 nm with an average equal 12.3 nm.

### Analysis of BET (brunauer-emmett-teller)

Adequate porosity and large surface areas are the two main factors that determine the effectiveness of nonmaterial. The results of the nitrogen adsorption test (BET) indicated that the synthesized nano-mullite exhibited a type IV-(a)-H3 hysteresis loop isotherm, which IUPAC-1985 defines for physisorption isotherms Supplementary Fig (S1). According to the BET analysis, the synthesized nano-mullite exhibited suitable mesoscale surface area and porosity, with an average pore size of 7.224 nm, 93.71 m^2^/g of surface area, and about 0.426 cm^3^/g of pore volume^27,46,52^.

### Contact angle

Mullite nanoparticles have been tested for hydrophilicity and hydrophobicity using contact angle measurements. It is known that the nano mullite has an average contact angle of 115.3°. This value, which is significantly higher than 90°, indicates that the produced nano mullite’s hydrophobic properties^27,46,49,53,54^. The important characteristics of nano mullite, such as its large surface area, hydrophobicity, and mesoporous structure, are revealed by its characterization. Because of these characteristics, it has many uses, including as a catalyst, adsorbent, and sensor. It can also help remove various heavy metals and dyes. It is possible to use the generated nano mullite to eliminate acid red 2 dye through adsorption.

### Data analysis and statistical techniques

In order to quantify the parameters as a percentage of removal inside the removal depending on adsorption (RDA) research, a mathematical model needs to be fitted to the data. The developments of the correlation model for the elimination % were carried out using the program build Expert® 10. Four input parameters as pH, adsorbent quantity, rpm, and contact time were used to construct a regression equation that models the output reactions. In an effort to attain a more exact and precise result, many tests have been conducted, modifying different components. ANOVA was used to check how well the model fit the data^43,44,55^. Also, to find out how important the model is, we ran a few experiments. There is an analysis of variance (ANOVA) for adsorption-based removal in Table 1. Nano-mullite that is produced

**Table 1:** ANOVA test for adsorption-based removal.

Source	Sum of Squar0es		Df		Mean Square			F- Value		p-value Prob > F			
Model	14,198.21		14		1014.16			19.37		< 0.0001		significant	
A-dosage	6079.78		1		6079.78			116.11		< 0.0001			
B-pH	325.63		1		325.63			6.22		0.0318			
C-TIME	293.73		1		293.73			5.61		0.0394			
D-rpm	1.02		1		1.02			0.020		0.8916			
BC	363.32		1		363.32			6.94		0.0250			
BD	341.99		1		341.99			6.53		0.0286			
B^2^	836.34		1		836.34			15.97		0.0025			
Residual	4.15		4		1.04								
Lack of Fit	4.15		3		1.38							Non significant	
Pure Error	0.000		1		0.000								
Cor Total	119.43		13										
Std. Dev	7.24		R-Squared					0.964					
Mean	27.51		Adj R-Squared					0.914					
C.V. %	26.31		Pred R-Squared					0.762					
PRESS	3492.77		Adeq Precision					15.868					
Response	Pred Mean	Pred Median	Obs	Std Dev		SE Mean	95% CI low		95% CI high		95% TI low		95% TI high
Removal	93.42	93.42	-	7.24		5.11	82.02		104.81		54.45		132.37

A 95% confidence level is used in the study. Every term in the model has its degree of significance indicated by the notation ‘P < 0.05’. The model and experimental findings are very congruent, as seen in Table 1. Coefficients of determination (R^2^ and Adj R^2^) were found to be more than 95%. The actual data is thought to fit well with the model if the value of R^2^ score is 1. The importance of considering the relevance of ‘lack of fit’ is vital when selecting a model^40^. A significant model is indicated by an F-value of 19.37. To have an F-value of this magnitude caused by noise is exceedingly unlikely (0.01%). Results with "Prob > F" values below 0.0500 are considered significant model terms. Here, we see the significance of the model terms A, B, and AB. "Pred R-Squared" and "Adj R-Squared" are reasonably congruent with each other, with a difference of less than 0.2. “Adeq Precision” reveals is the signal-to-noise ratio. When the ratio is more than 4, it is considered optimal. It is deemed sufficient to have a signal-to-noise ratio of 15.868. With this model, we may venture into the design area. The coded factor equation allows one to predict the response from the given elemental concentrations. Commonly, factors with high levels are recorded as +1 and those with low levels as −1 to assess the relative importance of these elements, it is helpful to compare their coefficients using the coded equation. As in Table 2, the value of dosage coefficient is higher than pH. The high value of +72.585 expresses the importance for gathering of these two factors (pH and dosage) and give them more attention through the process for maintaining the highest efficiency of the removal procedure. Lack of fit refers to how well the model can capture the relationship between different variables. Non-significant lacks of fit values indicate that the model fits the data well^56^.

**Table 2:** Final equation of actual factors that effect on the performance of removal.

Dosage	pH	TIME	Rpm	dosage * pH	dosage * TIME	dosage * rpm	pH * TIME	pH * rpm	TIME * rpm
−466.14	−50.37	+5.92	+0.14	+72.59	−1.35	+0.03	−1.03	−0.02	+0.002

**Fig. 5:**
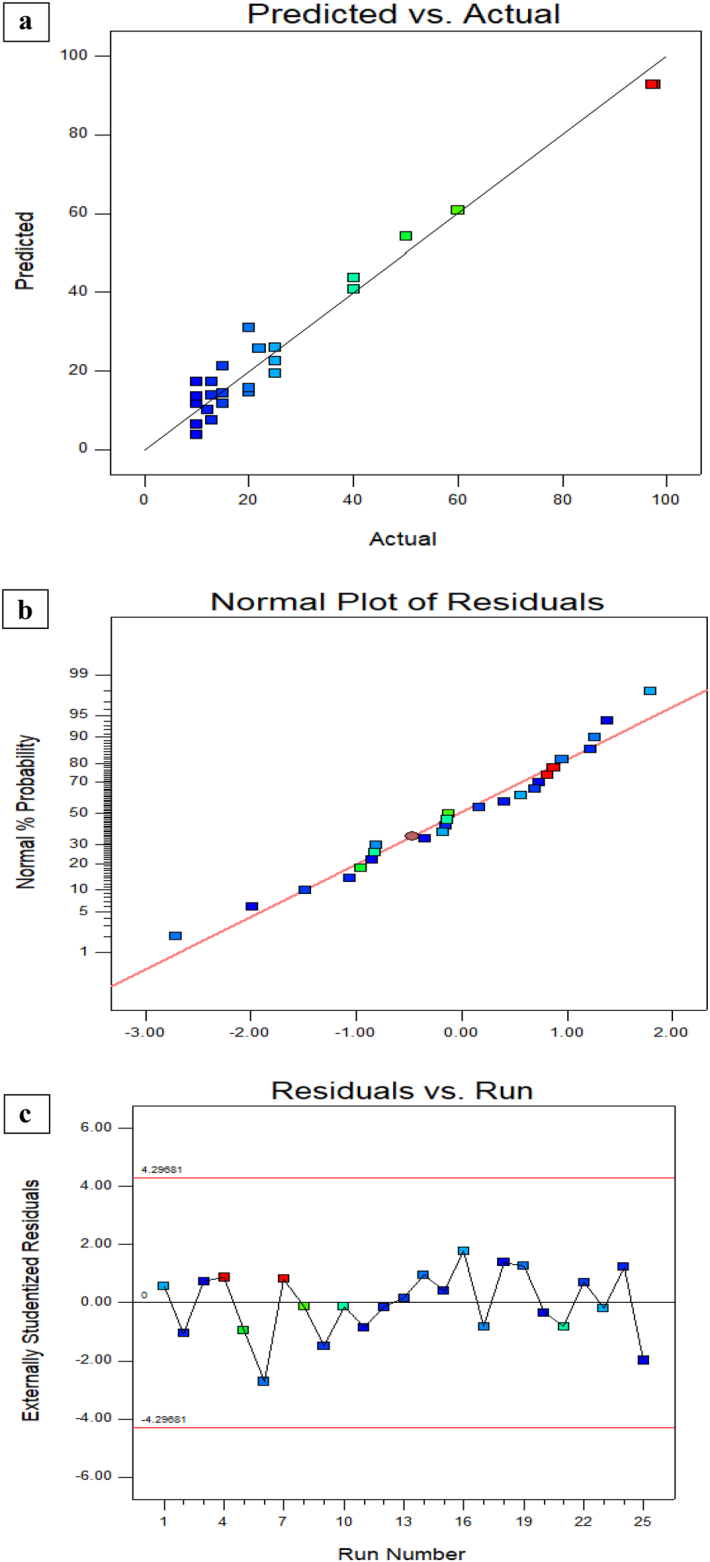
(a, b, c).Predicted against the actual values (**a**), Normalization plot (**b**), and Randomized distribution of error with respect run (**c**).

**Fig. 6:**
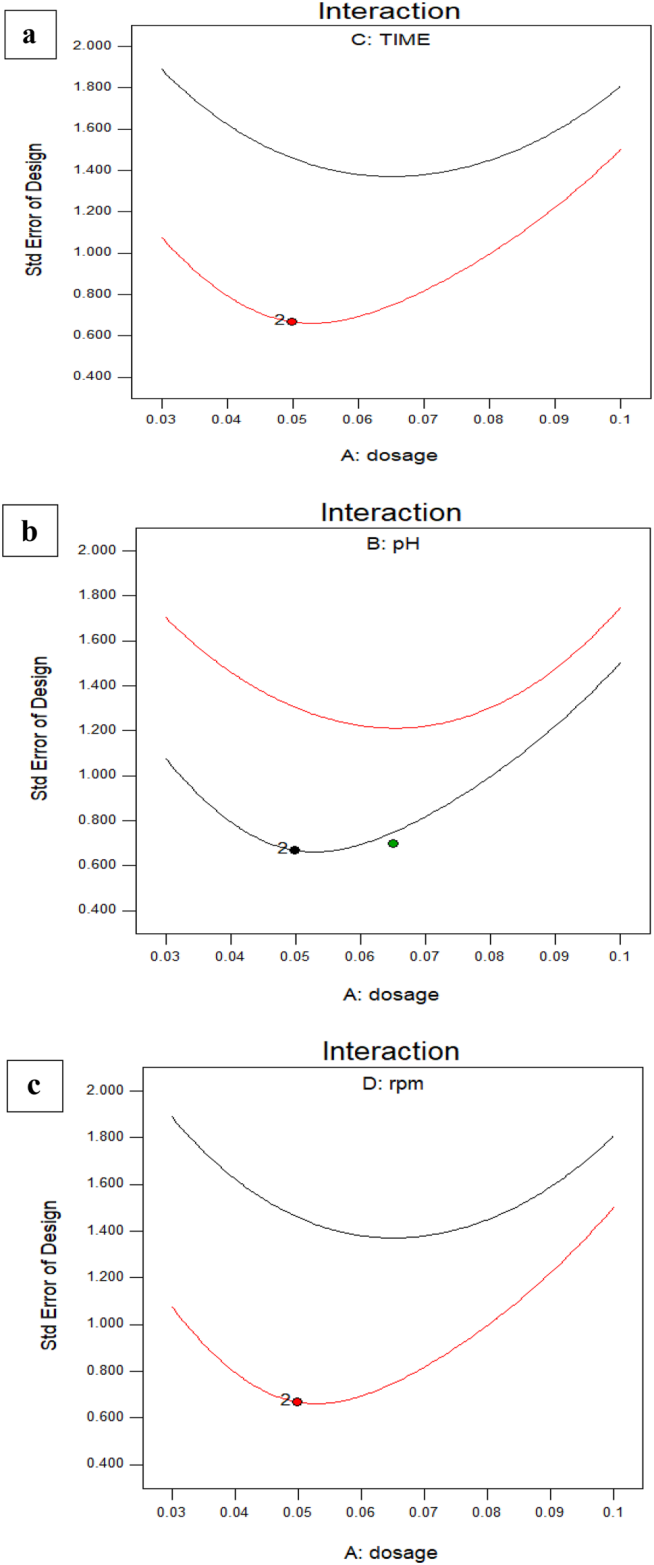
(a, b and c). Interaction between different factors (**a**), relation between dosage and time, b, the relation of dosage and pH, c, the interaction of rpm against dosage.

You may use this paradigm to find your way around the design area. A probability plot was used to evaluate the adequacy of the developed model. There is a statistically significant agreement between the predicted and actual values, as shown in Fig. 5a. The model seems to be appropriate given the presence of errors distributed linearly. There is a strong relationship between the projected results and the residuals. Fig. 5b again shows that the errors follow a normal distribution. The errors cluster in a linear fashion, as shown in the normal probability plot of residuals, proving this point as Fig. 5c. In a perfect world, there would be no outliers and the normal plot of residuals would display a linear trend. Precise line-to-data alignment is not required. “Fat pencil” test is the name of an effective guideline. The data may be deemed to appropriately follow a normal distribution if they can be entirely obscured by drawing a thick pencil line across the line. The strategy appears to be adequate in this case. Figure 6a shows that there is non-significant interaction between dosage and pH, also that the perturbation of the four factors which can effect on the removal % values as in Fig. 6b which explain that there is no interaction between dosage and time in case of this optimum case. Also Fig. 6c explains that there is no interaction between stirring rate and dosage. Dosage and pH are considered as the most effective parameters that can influence the adsorption % values. Supplementary Fig (S2) explains the perturbation of the most effective parameters on the adsorption of the dye and the relation between each other. It was linear relation between the dosage and the removal %; it increases with increment dosage of NPS on the contract the adsorption decreases with increment the pH^57,58^.

**Fig. 7:**
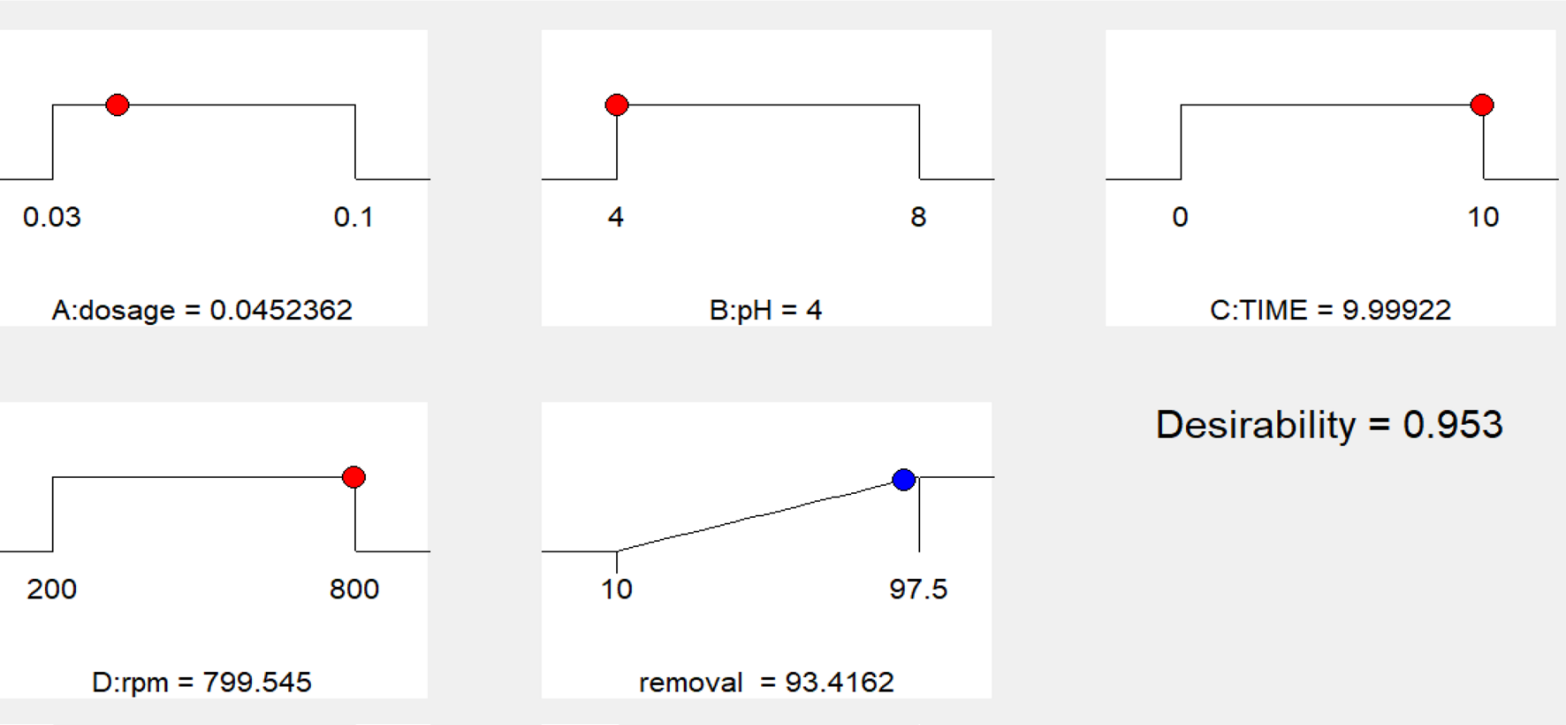
A ramp plot indicating the optimal combination of dose and contact time for optimum dye elimination and desired outcome.

**Fig. 8:**
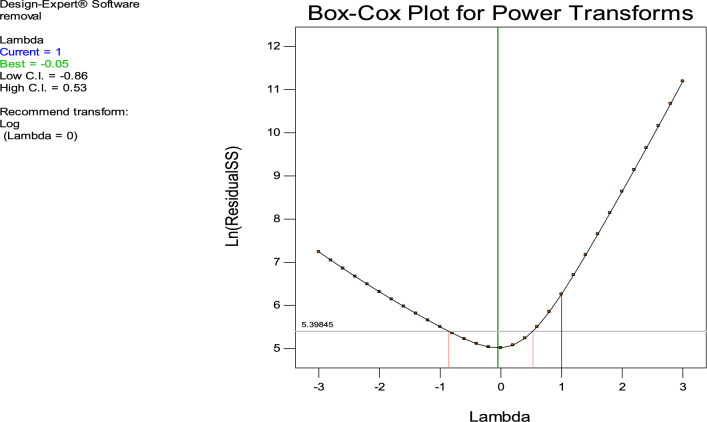
Plot of coxbox for removal efficiency.

**Fig. 9:**
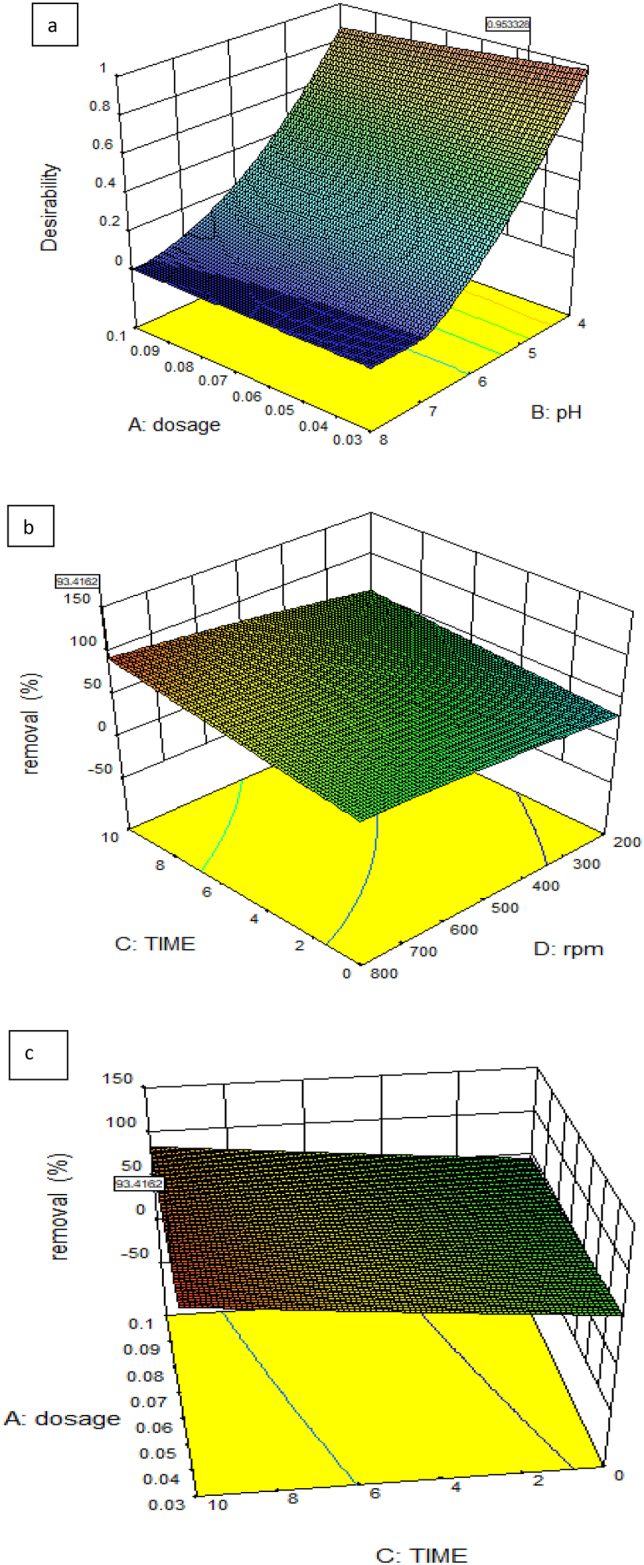
(a,b,c). 3D curves model of color removal using varying different parameters.

Optimizer variables with a high appeal level, approaching 1.00, are shown in Fig. 7 according to ramp plot which summarize the factors effecting the removal with their optimization for maximum desirability. You may use the Box-Cox plot, shown in Fig. 8, to figure out which power transformation is best for your response data. The power function is a good fit for describing most data transformations. The value of lambda (λ) that causes the modified model’s minimal residual sum of squares is shown by the lowest point on the Box-Cox plot. Positive reactions are the only ones that may undergo power law transformations. Before looking for the optimal power, a constant, k, is introduced to make sure this condition is true. You can see the best predicted λ, the confidence interval around it, and the presently applied λ on the figure. It is because they do not fall within the 3 lambda limits that the confidence interval bounds are not displayed. In order to demonstrate that the model is adequate without resorting to power transformation, the program will propose a standard transformation that is within the confidence interval and comes closest to the optimal lambda value. Figure 9 shows the three-dimensional graphs that show different parameters that targets high removal % values and the maximum desirability. Fig. 9a; explain the relation between the dosage of nonmaterial and pH, Fig. 9b is the relation between the time and stirring rate (rpm) and Fig. 9c shows the relation between dosage and the contact time. The following equation express the RSD model, at 800 rpm the only variables were dosage time and pH. The equation was Removal=+98.23 +1.62*A +0.012* B+0.24* C+0.12* AB+0.44* AC-0.76* BC-2.42* A^2^−3.60* B^2^−1.81* C^2^

## Using the batch method

### pH impact

**Fig. 10:**
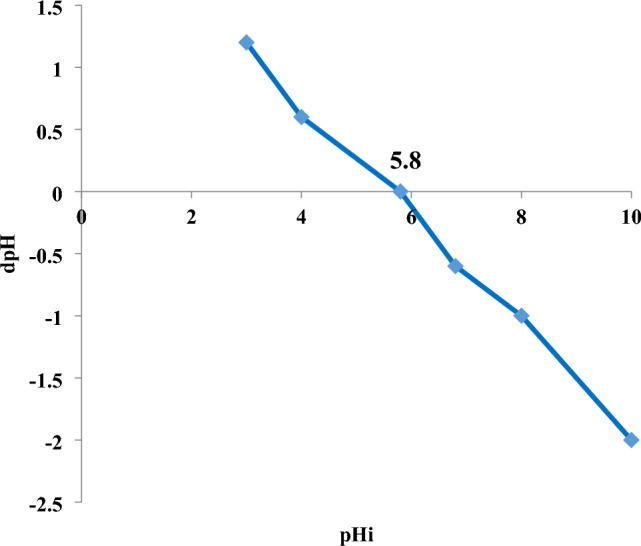
Zero point charge measurement of mullite nanoparticles.

**Fig. 11:**
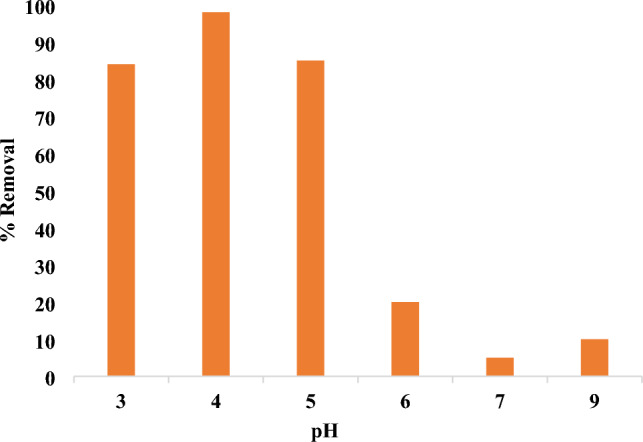
pH effect on the removal of MR with dose 0.05 g of nano mullite in 10 min.

[H]^+^ concentration is a crucial parameter that influences both interfacial transport phenomena and the surface charges of the adsorbent, hence impacting the removal procedure^59^. The solution is basic if pH is more than 7.0 but it is acidic in case of pH is less than 7.0. The pH of the aqueous medium will consequently influence the adsorption rate; thus, the quantity of electrostatic charges transferred by the ionized dye molecules is contingent upon the medium’s pH. Fig. 10 illustrates that the pH_pzc_ of mullite nanoparticles is 5.8, indicating that the charge of the surface is positive below this value and above it, it is negative^60^. The adsorption process of nano mullite with the anionic dye (methyl red) is enhanced in an acidic solution due to the positive charge of mullite and the presence of a carboxylic group in methyl red, leading to electrostatic interactions between them^61^. Fig. 11 illustrates that at pH 4.0, the percentage removal reached approximately 98% with an adsorbent dosage of 0.05 g/100 mL with 10 ppm of methyl red dye after 10 min. It demonstrated a gradual decline, reaching approximately 20% at pH 6.0 and 10% at pH 9.0, primarily due to the carboxylic group was deprotonated^61^.The dye acquired a negative overall charge, resulting in repulsion by the anionic mullite nanoparticle at pH levels higher than 5.8.

### Dosage of adsorbent impact

**Fig. 12:**
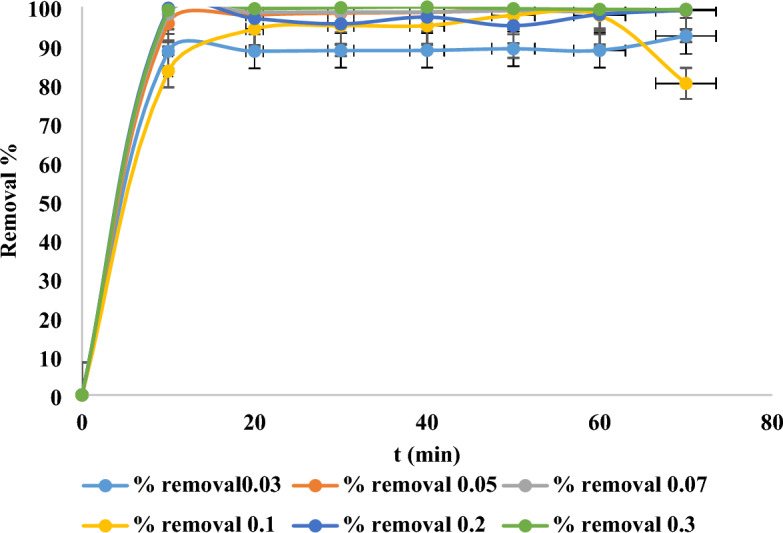
Doses of adsorbent effect on 10 ppm elimination of MR dye and nano mullite.

By creating an adsorbent-adsorbate solution, various amounts of adsorbents were added to a constant dye concentration, one can test the impact of adsorbent dosage on the adsorption process. At different dosages, the impact of adsorbent dosage on the elimination of MR dye was examined (0.03, 0.05, 0.07, 0.1, 0.2, 0.3 g/100 ml) while keeping additional constant variables. It was found that when the dosage of adsorbent was increased, the removal efficiency of MR increased as well. The rise in removal percentage could be caused by the improvement of the adsorbent’s surface and quantity of dye adsorption sites^62–64^.As shown in Fig. 12, the dye’s adsorption increased quickly until the optimum dose. All amounts above 0.05 g can give a significant removal more than 98% in the 10 min. as 0.07 g which gave 99%. The optimum dose was chosen according to the lowest number of nanoparticles that gave a significant removal within short time so it will be at 0.05 g which give about 98% in the first 10 min.

### Stirring rate impact

**Fig. 13:**
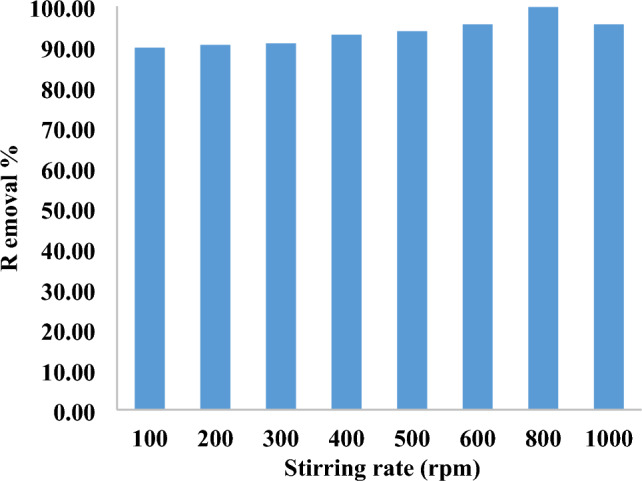
Stirring rate effect on 10 ppm elimination of MR dye with dose 0.05 g of nano mullite.

The rate of adsorption is influenced by the agitation speed, as shown in Fig. 13. The removal of dye was followed at different stirring rates between 100 and 1000 rpm at pH=4 at room temperature within a concentration of 10 ppm, and the dose of adsorbent of 0.05 g/100ml. The percentage of removal elevated from 89.23 to 99.34 when the rate of stirring increased from 100 to 1000 rpm. So, it is clear that the stirring rate has the lowest effect on the adsorption process because in all ranges the removal % was near 90% or more. This could be explained by the fact that movement increases the system’s mobility and lowers the boundary-layer resistance. Additionally, the speed of the agitation affects the impact of external mass transfer, which encourages close contact between the adsorbent and adsorbate phases^65^. The best speed that scored high adsorption was at 800 rpm, where the removal percentage reached 98%.

### Contact time impact

**Fig. 14:**
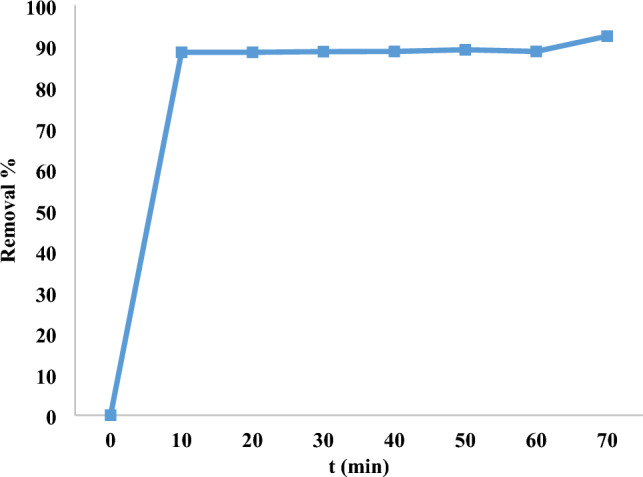
Contact time impact on 10 ppm elimination of MR dye with dose 0.05 g of nano mullite.

A crucial component of an economical treatment of wastewater system is time of equilibrium. At the starting dye concentration of 10 ppm, the impact of contact duration on dye adsorption was examined over a range of time intervals, from 0 to 70 min. The impact of contact time on the removal percentage of MR dye was examined, as illustrated in Fig. 14. At first, the proportion of dye removed by nano mullite slowly, but it gradually climbed until it reached equilibrium and reached 98% in 10 min using 800 rpm. Due to the nano mullite’s available adsorption sites, the amount of colors adsorbed increased rapidly and accessible pores, which progressively fill up and slow down adsorption until it reaches complete saturation^46^.

### Initial dye concentration impact

**Fig. 15:**
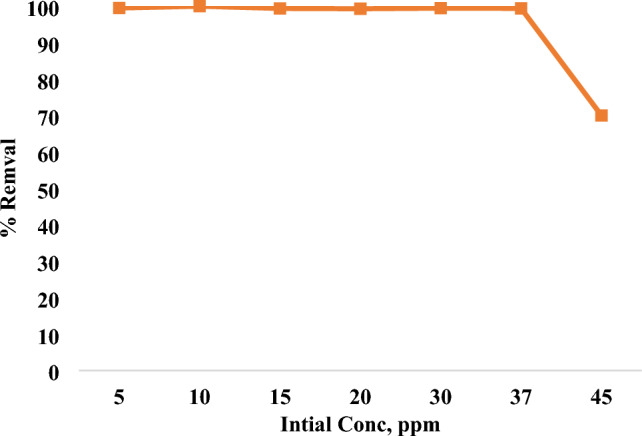
Concentration impact on elimination of MR dyes with dose 0.05 g of nano mullite.

Dyes accumulate at the interface between the liquid and solid phases, which is a mass exchange process (dye adsorption). Dye concentration at start-up provides the necessary push to overcome mass transfer barrier from liquid to solid. The driving force improves with increasing initial focus^66^. As seen in Fig. 14, it shows how the initial concentration of MR dye affects the percentage of dye removed from different adsorbents. Different initial MR concentrations (5, 10, 15, 20, 25, 30, 37 and 45 ppm) were used for the experiments, as the concentration increased, the rate of removal increased. The best removal efficiency was at 37 ppm. So, the optimum condition for the removal of 98 % of MR dye was pH=4, 800 rpm, 0,05 g of nano mullite in 10 min, in addition to the ability to remove up to 37 ppm of MR dye, as shown in Fig. 15, where the removal % decreases at 45 ppm.

### Adsorption isotherm

The optimization of the adsorption system relies heavily on adsorption isotherms. The maximal adsorption capacity and the equilibrium connection between the adsorbent and adsorbates may be explained by the adsorption isotherm. The adsorption isotherm was employed to measure the adsorbent-adsorbates interaction^67^. The fitting analysis of the experiment results made use of Langmuir, Freundlich, Temkin, and DKR models.

### The langmuir isotherm

When the adsorbate was uniformly distributed over the adsorbents in a monolayer coverage experiment, the Langmuir isotherm model was commonly employed^68^. It is as:

3 $${\text{q}}_{\text{e}}={\text{q}}_{\text{max}}*\frac{{\text{bc}}_{\text{e}}}{1+{\text{bc}}_{\text{e}}}$$

Through the linearization of Eq.3 it becomes:

4 $$\frac{{\text{c}}_{\text{e}}}{{\text{q}}_{\text{e}}}=\frac{1}{{\text{q}}_{{\text{max}}^{*\text{b}}}}+\left(\frac{1}{{\text{q}}_{\text{max}}}\right){\text{c}}_{\text{e}}$$

**Fig. 16:**
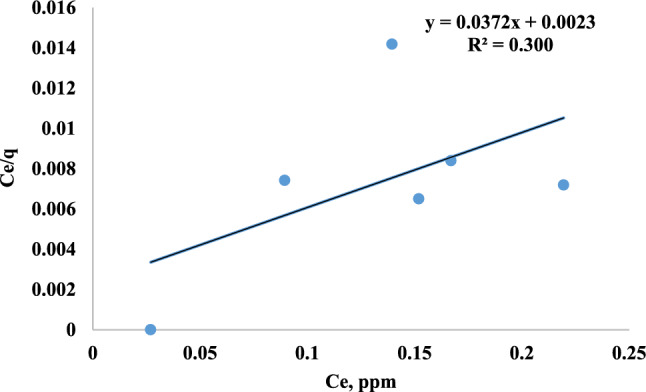
Langmuir isotherm of MR adsorption.

Where q_e_ is the amount of MR sorbed per unit mass of adsorbents (mg/g) and C_e_ is the MR equilibrium concentration in the solution (ppm). The monolayer sorption capacity (mg/g) and the Langmuir equilibrium constant (L/mg) are represented by the Langmuir constants q_max_ and b. Fig. 16 and Table 3 illustrates the Langmuir isotherm, which plots C_e_/q_e_ against C_e_, resulting in a straight line with a slope of 1/q_max_ and an intercept of 1/b*q_max_. The Langmuir constant equilibrium (b) was 16.174 L/mg and the measured concentration of maximum adsorption capacity of MR (q_max_) was 26.88 mg/g. The R^2^ value =0.300 which is too low to be used as discretion of the model.

**Table 3:** Langmuir, freundlich, DKR, temkin and R^2^ values of MR adsorption on mullite nanoceramic material.

Adsorption Isotherm	Values
Langmuir	R^2^= 0.300 q_max_=26.88 mg/g b=16.174 L/mg
Freundlich	n=1.19 k_f_=89.76 (mg/g) (L/g)^1/*n*^ R^2^=0.830
DKR	E= 3.10 kJ/mol qmax=68.79 mg/g R^2^= 0.945
Temkin	b=236.4 J/mol A=44.63 L/mg R^2^= 0.662

The constant dimensionless separation factor R_L_ is produced by computing the Langmuir constant equilibrium (b) and the Cˈ initial concentration of MR in the solution, as shown in Eq. 5.

5 $${\text{R}}_{\text{L}}=\frac{1}{1+{\text{bC}}_{\text{o}}}$$

There is an explanation of the shape and favorable adsorption between MR and adsorbents by R_L_. The value of R_L_ can be used to determine the favorable (0 <R_L_<1), unfavorable (R_L_>1), linear (R_L_ = 1), or irreversible (R_L_ = 0) shape of the isotherm^69^.The ${\text{R}}_{\text{L}}$ equal 0.0227, indicating favorable adsorption.

### The freundlich model

The Freundlich model describes MR adsorption as a multilayer process characterized by heterogeneity. According to the theory, stronger binding was occurred at the most heavily occupied binding sites and weakens as the number of sites occupied grows. The model’s expression could be shown as follows:

6 $${\text{q}}_{\text{e}}={\text{K}}_{\text{f}}{\text{C}}_{\text{e}}^{1/\text{n}}$$

The following constants and logarithms can be used to make this expression linear:

7 $${\text{logq}}_{\text{e}}={\text{logk}}_{\text{f}}+\frac{1}{\text{n}}{\text{logC}}_{\text{e}}$$

where 1/n is the adsorption intensity, which is related to the heterogeneity of the adsorbent surface, n; indicates favorability and K_f_ Freundlich constants is the adsorption capacity (mg/g) (L/g)^1/*n*^^70^. Whereas the concentration-dependent increase in adsorption becomes less pronounced at higher concentrations and lower ones, respectively. When the K_f_ value is higher, the adsorption intensity is also higher.

**Fig. 17:**
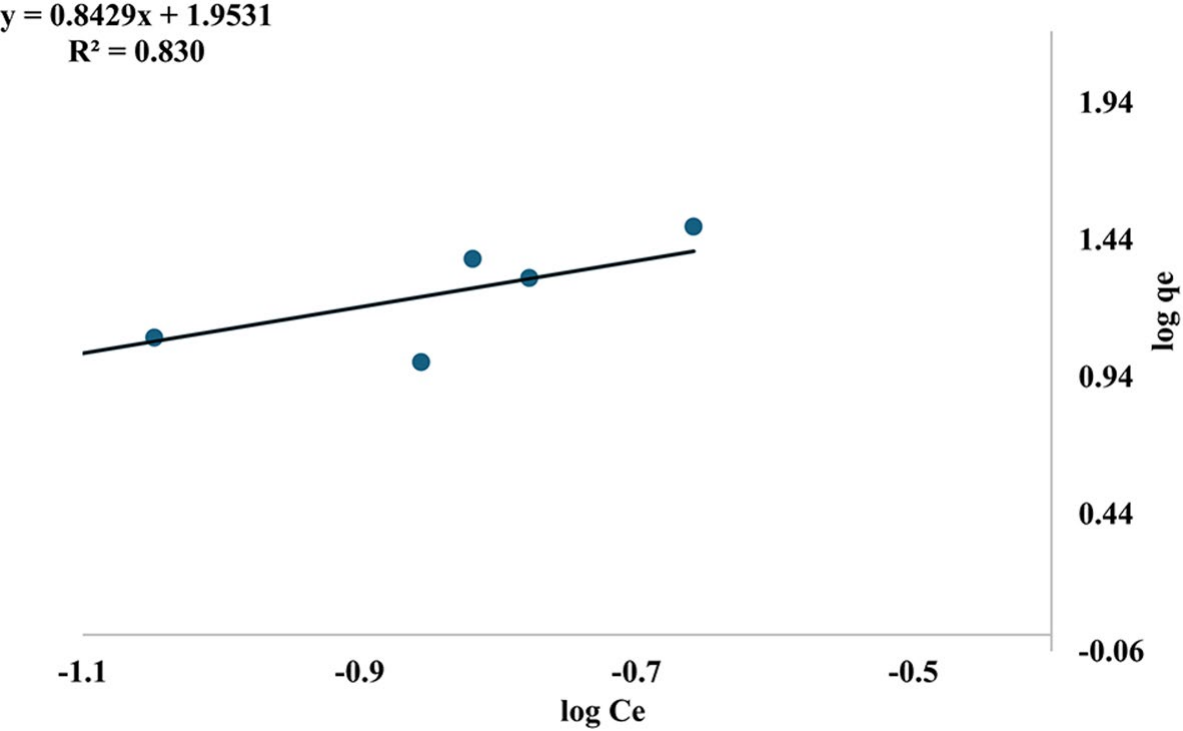
Freundlich isotherm of MR adsorption.

Table 3 and Fig. 17 illustrate that the Freundlich isotherm indicates k_f_ = 89.76 (mg/g) (L/g) and n = 1.19, which exceeds 1. This suggests that the physical adsorption process is favorable; conversely, an n value less than one would imply a chemical adsorption process. However, if the value of n exceeds one, adsorption is identified as a physical process^71,72^. Additionally, R^2^ = 0.830 is insufficient for explaining the model.

### Dubinin-kaganer-raduskevich (DKR)

Since it does not presume a homogeneous surface or constant adsorption potential, the more thorough empirical model known as the Dubinin-Radushkevich isotherm outshines the Langmuir isotherm^71^. The process of MR adsorption on that material is outlined here. The pore-filling process is the main point of emphasis. This technique allows molecules to be released from their sorption positions to an endless distance, and it distinguishes between physical and chemical adsorption^57,73^. The isotherm, which has the following linear form, is used to find the apparent energy of magnetic resonance adsorption onto an adsorbent^74^.

8 $${\text{lnq}}_{\text{e}}={\text{lnq}}_{\text{max}}-\upbeta {\upvarepsilon }^{2}$$

9 $$\upvarepsilon =\text{RTln}\left(1+\frac{1}{{\text{c}}_{\text{e}}}\right)$$

Where q_max_ indicates the maximum sorption capacity (mg/g), ε stands for the Polanyi potential, and β is an activity coefficient constant related to sorption energy. The following illustrates how each sorbate molecule’s average free energy (E) of sorption traveled from infinity in the solution to the solid surface as shown in the following Eq.10^75^.

10 $$\text{E}=\frac{1}{\sqrt{2\upbeta }}$$

**Fig. 18:**
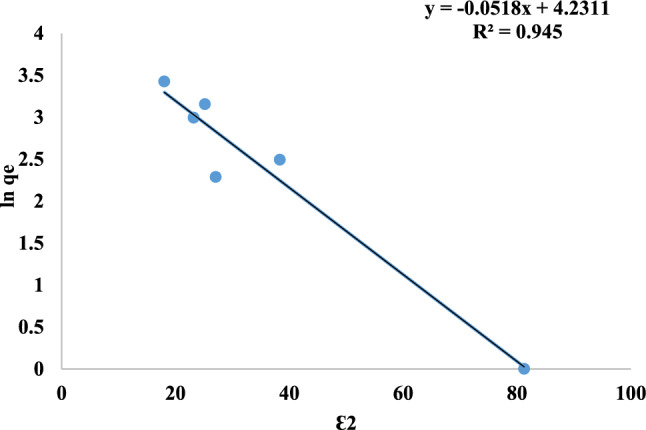
DKR isotherm of MR adsorption.

The nature of the adsorption process is primarily determined by the mean free energy (E). Specifically, when 8 < E < 16 kJ/mol, the process is classified as ion exchange; if E is less than 8000J/mol, it is characterized as physical interaction; and if E more than 16000 J/mol, it is identified as chemical interaction. Figure 18 and Table 3 demonstrate that straight lines were generated when the DKR isotherm was plotted with ln q_e_ against ɛ^2^. The value of E which calculated using equation 10, is 3.10 kJ/mol, that indicating that the physical interaction. The R^2^ results indicate that the DKR isotherm model is more appropriate for the adsorption of MR dye onto Nps, with a high correlation coefficient of R^2^ = 0.945^58,73^.

### The temkin isotherm

We may highlight this isotherm by assuming two things. It begins with the premise that, as a result of interactions between the adsorbent and the adsorbate, the adsorption heat of the whole molecular layer decreases linearly with increasing coverage. In addition, the adsorption is characterized by a uniform distribution of binding energies that is close to the maximum binding energy. If the sorption heat distribution is not uniform, the Temkin isotherm is a popular tool to utilize^76^.

11 $${\text{q}}_{\text{e}}=\frac{\text{RT}}{\text{b}}\text{lnA}+\frac{\text{RT}}{\text{b}}{\text{lnC}}_{\text{e}}$$

**Fig. 19:**
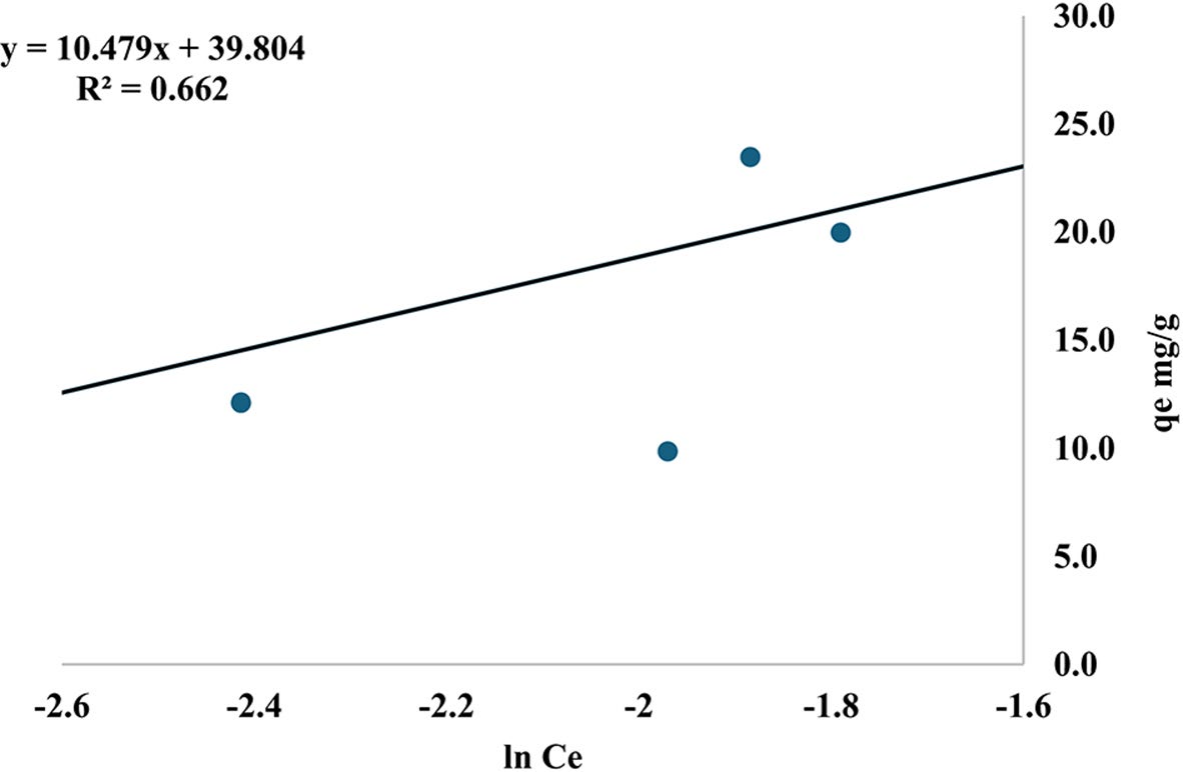
Temkin isotherm of MR adsorption.

A (L/mol) the equilibrium binding constant which represents the highest possible binding energy. In the Temkin plot (q_e_ against ln C_e_) as Fig. 19, the heat of sorption (J/mol) is calculated from the relationship between RT and the sorption constant (b), and the universal gas constant (8.314 J.mol) is denoted by R. The adsorption heat constant is B. T= 298 K (or 25 °C) is the temperature^77^, b = 236.4 J/mol, A = 44.63 L/mg and R^2^ = 0.662. There seems to be little interaction between MR and nano mullite, as the value of b has decreased. One way to describe the process of MR adsorption onto nano mullite is as physisorption. There is evidence that the ion-exchange process has a bonding energy range of 8–16 kJ/mol. Adsorption energies that are lower than this range are, on the other hand, shown by physisorption processes^71^. R^2^ for this isotherm equals 0.662 also this cannot fit the model.

The experimental data from the isotherm (Table 3) shows that the Langmuir equation provides the worst fit, as shown by the lowest correlation coefficient value of 0.300. With a value of 0.945, the DKR model outperforms the other models that were considered. It can be concluded that the model well describes the MR adsorption onto nano mullite.

## Adsorption kinetics

The intra-particle diffusion kinetics and pseudo-first and pseudo-second order MR dye removal kinetics have been used to study the kinetics of MNC.

The following are the adsorption kinetics equations:

12 $$\text{log}\left({\text{q}}_{\text{e}}-{\text{q}}_{\text{t}}\right)={\text{logq}}_{\text{e}}-\left(\frac{{\text{k}}_{1}}{2\cdot 303}\right)\text{t}$$

13 $$\frac{\text{t}}{{\text{q}}_{\text{t}}}=\left(\frac{1}{{\text{k}}_{2}{\text{q}}_{\text{e}}^{2}}\right)+\left(\frac{1}{{\text{q}}_{\text{e}}}\right)\text{t}$$

Where equations 12 and 13 represent the pseudo-first order and pseudo-second order, respectively. The adsorption rate constants of the pseudo-first order and pseudo-second order expressions are k_1_ (min^−1^) and k_2_ (g/mg.min), respectively; the quantities of MR adsorbed (mg/g) at time t and equilibrium are q_t_ and q_e_^77,78^.

**Fig. 20:**
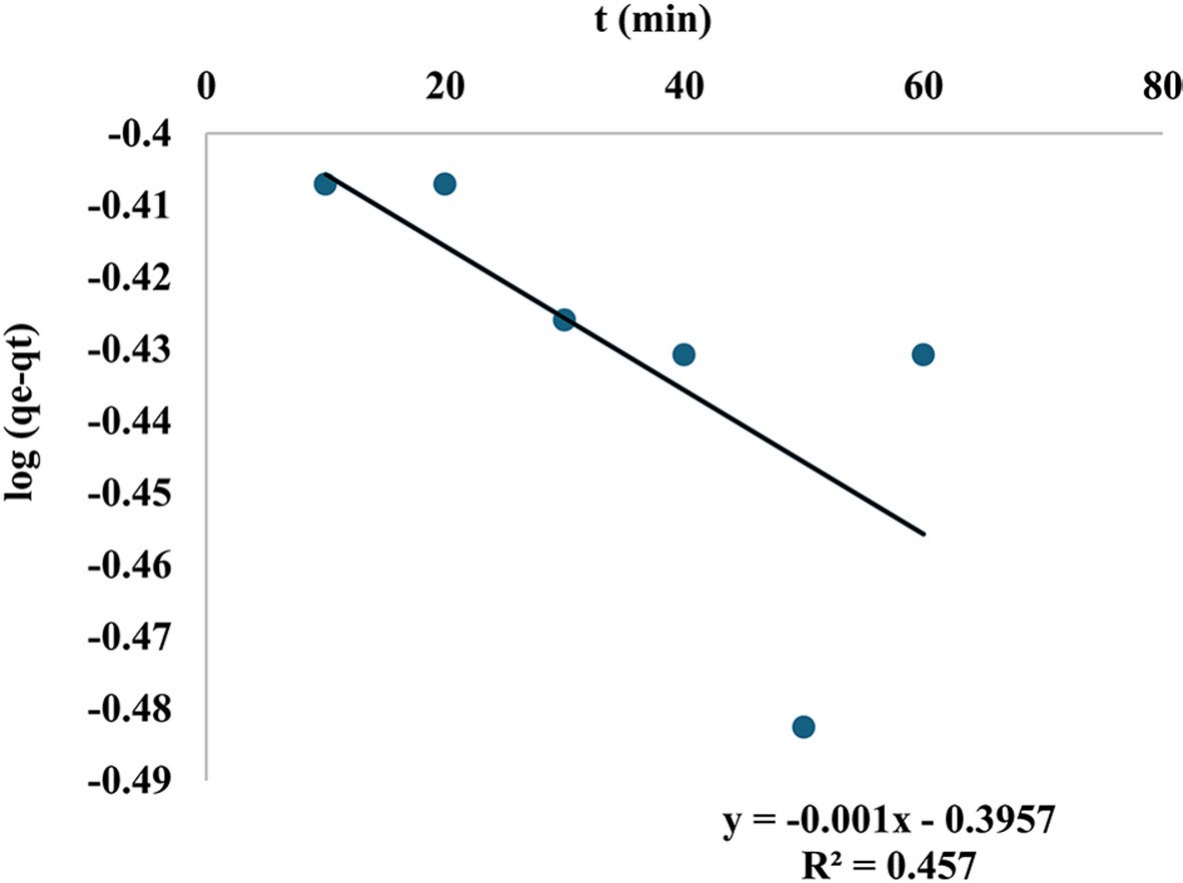
Pseudo-first order of MR adsorption.

**Fig. 21:**
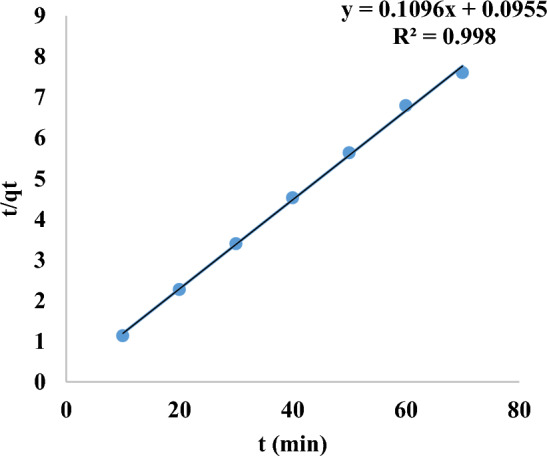
Pseudo-second order of MR adsorption on mullite nanoceramic material.

Figure 20 and Table 4 illustrate that plotting log (q_e_-q_t_) against time for the Pseudo-First-order model reveals a linear relationship, with an R^2^ value of 0.457 and a rate constant k_1_ of 0.022 min^−1^. In Fig. 21, plotting t/qt against t yields a straight line with a R^2^ value of 0.998, and a rate constant k_2_ of 0.126 g/mg.min for the pseudo-second-order model. The pseudo-second-order model is the most suitable for the removal of MR, evidenced by its high regression R^2^ value of unity and the value of the q_e_ cal is almost equal to q_e exp_. The adsorption process is contingent upon the quantity of nanomaterial and dye present.

**Table 4:** Adsorption kinetics of methyl red dye adsorption on mullite nanoceramic binding material.

Adsorption kinetics	Results
Pseudo-first order	k_1_ =0.002 min^−1^ qe exp.=9.321 mg/g qe cal.=0.364 mg/g R^2^ = 0.457
Pseudo-second order	q_e_ cal.=9.124 mg/g q_e_ exp.=9.321 mg/g k_2_=0.126 g/mg.min. R^2^ = 0.998.
Intra-particle diffusion	R^2^ = 0.389 k_i_ = 0.05 mg/g. min^1/2^ C = 8.59 mg/g

### Intra-particle diffusion

Intraparticle diffusion is a good tool to determine the process of methyl red adsorption on nano mullite. It states that the movements of methyl red dye on mullites’ pores affect the adsorption rate. This application shows that the diffusion process of methyl red dye on the pores of nano mullite is an important process.

The following equation is used to calculate intra-particle diffusion:

14 $${\text{q}}_{\text{t}}={\text{k}}_{\text{i}}{\text{t}}^{0.5}+\text{C}$$

**Fig. 22:**
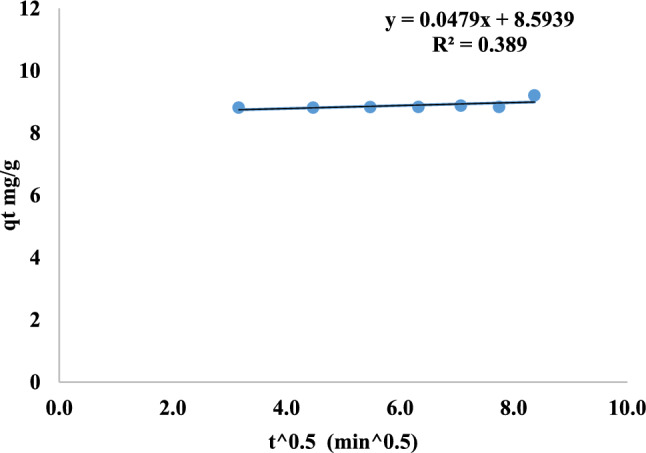
Intra-particle diffusion of MR adsorption on MNPs.

Where, the quantities of MR adsorbed (mg/g) at time t and equilibrium are q_t_, and the intra-particle diffusion rate constant is k_i_ (mg/g. min^1/2^). C: the thickness constant of the boundary layer (mg/g)^77,78^, as shown in Fig. 22 k_i_= 0.05 mg/g. min^1/2^, C = 8.59 mg/g, R^2^ = 0.389^79^. So, the rate determining step is diffusion boundary layer.

Table 4 showed that adsorption kinetics of this removal would follow pseudo-second-order kinetics which depends on the concentration of both adsorbent and adsorbate.

## Thermodynamic study

**Fig. 23:**
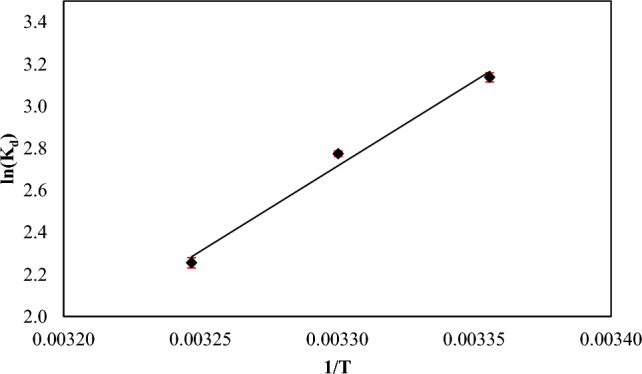
Van’t Hoff plot of methyl red dye adsorbed onto nano mullite.

Thermodynamic parameters are employed to evaluate the spontaneity of an absorption process. The alterations in free energy (ΔG°), enthalpy (ΔH°), and entropy (ΔS°) of the absorption process was ascertained utilizing the subsequent equations(15-17):

15 $${\text{K}}_{\text{d}}=\frac{{\text{q}}_{\text{e}}}{{\text{C}}_{\text{e}}}$$

16 $$\Delta {\text{G}}^{\text{o}}=-{\text{RTlnK}}_{\text{d}}$$

17 $${\text{lnK}}_{\text{d}}=\frac{-(\Delta {\text{H}}^{\text{o}})}{\text{RT}}+\frac{\Delta {\text{S}}^{\text{o}}}{\text{R}}$$

q_e_ denotes the equilibrium concentration of the adsorbed dye on the adsorbent, whereas C_e_ indicates the equilibrium concentration of the residual dye in the solution. A linear relationship is the evident of Van’t Hoff plot of ln k_d_ versus the reciprocal of temperature (1/T) as shown in Fig. 23. The values of ΔH° and ΔS° can be determined by calculating the slope and intercept of the line. The ΔH° value of −67.217 kJ/mol indicates that the absorption of methyl red dye onto nano mullite is an exothermic process. The negative ΔS° value of −0.199 kJ/mol. K signifies a reduction in randomness at the absorption/solution interface during the dye absorption onto nano mullite. The ΔG° values at temperatures of 298, 303, and 308 K were −7.774, −6.988, and −5.776 kJ/mol, respectively, indicating that the absorption of methyl red dye is a spontaneous process^57,80^.

## Experiments on desorption and reuse

**Fig. 24:**
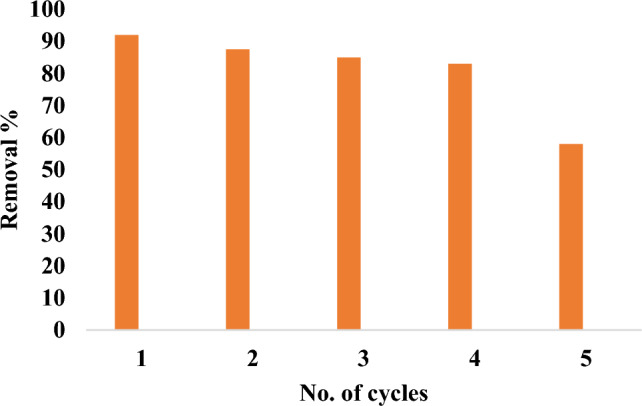
Recycle of nano mullite.

Four cycles of the adsorption-desorption research were made at room temperature. The experiments were conducted with a 1:1 ratio of NaOH to HCl. Following filtration and distilled water wash, the quantitative desorption of MR from mullite was accomplished. It is reusable several times. Fig. 24 illustrates that up to 4 cycles, more than 80% of the desorption process was successful in the fifth cycle the removal reach 58%. Mullite’s capacity to be reused illustrates the adsorbent’s affordability, practicality, and potential for assessment and use in wastewater treatment^81^.The decrease of the removal % in the recycle process may be due to loss some amount of the nanomaterial during washing, filtration and drying.

## Comparative studies

Table 5 shows the comparative study about the adsorption removal of methyl red using different adsorbent dosage, pH, contact time, with their adsorption capacity. As the following data the mullite NPS can give high adsorption capacity refer to the adsorbent dose 0.05g through just 10 min with removal about 99%.

**Table 5:** Comparison the previous studies.

Adsorbent	q_max_ [mg/g]	pH	Dosage g/l	Time	Ref
Calix[4]arene modified silica resin	154.6	6.6	0.4	-	^82^
Polya(azomethinethioamide) resin	90.9	6	0.4	-	^83^
Polyehyleneimine modified activated carbon	526	-	0.2	40 min	^7^
Amberlite IRC-50	5.08	8.5	1	120 min	^50^
BPA-PEA	49	6	2	18 h	^84^
Mullite NPs	68.79	4	0.5	10 min	This study

## Conclusion

In this study, we show that the synthesized MNPs material can effectively remove methyl red dyes from water-based solutions. Scientists looked at how variables such starting dye concentration, temperature, adsorbent dose, duration, pH, and shaking impact affected dye adsorption. With a starting dye concentration of 35 ppm at pH 4, after 10 min of contact time at room temperature and 800 rpm stirring speed, the produced mullite nanoceramic material was shown to be an effective MR dye adsorbent, with a removal efficiency of 98%. The adsorption experimental findings were used to fit the DKR isotherm model into the pseudo-second order model. The desorption study of MR dyes demonstrated the composite’s outstanding regenerative efficiency using 0.01mol L^−1^ HCl &0.01 mol L^−1^ NaOH reach to 4 cycles with more than 80% removal. All of those characteristics are good evidence which prove that mullite nanoceramic material can be used in the treatment of wastewater. The statistical approach successfully modeled methyl red adsorption onto mullite nanoparticles, identifying pH and adsorbent dose as most critical parameters. The developed model can guide practical wastewater treatment applications with high prediction accuracy. Future work was the investigation of adsorbent regeneration, application to real industrial effluents and life cycle assessment of the adsorption process.

## Supplementary Information

Supplementary Information.

## Data Availability

All data generated or analyzed during this study are included in the published article. Additional details regarding the experimental apparatus and materials are provided in the supplementary file.
