# Supplementary material for: ANOVA and design expert model for discoloring of azo benzene derivative dye used mesoporous aluminum silicon oxide nanoparticles
Source: Sci Rep. 2025 Dec 20;15:44302. doi: 10.1038/s41598-025-27886-x (PMC12722226; doi:10.1038/s41598-025-27886-x)
Supplement: Supplementary file 1 — Supplementary Information. [file 41598_2025_27886_MOESM1_ESM.docx]

**ANOVA and Design Expert Model for Discoloring of Azo Benzene Derivative Dye Used Mesoporous Aluminum Silicon Oxide Nanoparticles**

Yara M. Adly^1^, Magdi E.A. Zaki^3^*, Omar A. Fouad*^1^, Gehad G. Mohamed^1,2^, Sami A. Al-Hussain^3^, Maysa R. Mostafa^1*^

1. Chemistry Department, Faculty of Science, Cairo University, 12613, Giza, Egypt.
2. Nanoscience Department, Basic and Applied Sciences Institute, Egypt-Japan University of Science and Technology, New Borg El Arab, Alexandria, 21934, Egypt
3. *Department of Chemistry, Faculty of Science, Imam Mohammad Ibn Saud Islamic University (IMSIU), Riyadh 11623, Saudi Arabia*

**The corresponding authors**

***M.R. Mostafa:** e-mail: [maysaramadan140@cu.edu.eg](mailto:maysaramadan140@cu.edu.eg)

* M.E.A. Zaki: e-mail: [mezaki@imamu.edu.sa](mailto:mezaki@imamu.edu.sa)

*Omar A. Fouad : [oahmed@sci.cu.edu.eg](mailto:oahmed@sci.cu.edu.eg)

1. **Materials**

**2.1. Materials and solutions**

At the analytical level, every component was utilized exactly as intended. Aluminum chloride hexahydrate (purity 99%), tetraethyl orthosilicate (purity 98%), and ethyl alcohol are from Sigma-Aldrich Chemie GmbH, which is based at Eschenstrasse 5 D-82024 TAUFKIRCHEN. The ammonia solution came from the German company Riedel-deHaen. The source of hydrochloric acid (HCl) and sodium hydroxide (NaOH) (purity 95%) were Honeywell, Germany. The methyl red dye (purity 95%) was bought from Sigma-Aldrich and used just as it was delivered, requiring no further processing. Stock solutions of methyl red dye were prepared using distilled water. Dilution allowed for the achievement of the required concentrations.

1. **Instruments**

Using a Bruker D8 Discover X-ray diffractometer, the phase composition of the synthesized nanoparticles and sintered samples was ascertained using Ni-filtered Cu K radiation (= 1.5406). N2 has been employed as the adsorptive gas in gas adsorption investigations in order to calculate the BET surface area at 77 K. The materials were evacuated under a high vacuum for four to twelve hours prior to the adsorption test. The Brunauer-Emmett-Teller (BET) hypothesis served as the basis for the computation, and a Nova Touch LX2 analyzer was used for the study. The microstructure and pore size distribution of a  selected samples were examined using a scanning electron microscope (SEM) of cracked surfaces utilizing the Philips XL30 model, an accelerating voltage of 30 kV, magnification up to 400000, and resolution for W [3.5 nm]. Samples were lightly coated in gold prior to testing. Transmission electron microscopy was used to analyze the size and form of the produced nanoparticles (TEM; JEOL JEM-2100, Tokyo, Japan). The quantities of the dyes under study were determined using a spectrophotometric method. Plotting the dye's absorbance versus concentration at a maximum wavelength of 520 nm for methyl red dye allowed for the creation of calibration curves using a UV-vis spectrophotometer. The final dye concentration was determined spectrophotometrically in accordance with the dye λmax using the Beer- Lambert equation.

Supplementary Table S1 : XRD data and crystalite size of the nanomullite

| 2θ | hkl planes | FWHM (in radians) | crystallite sizes (nm) |
| --- | --- | --- | --- |
| 31.06° | (0 0 1) | 1.12 | 7.33 |
| 33.07° | (2 2 0) | 0.90 | 9.19 |
| 36.84° | (1 3 0) | 1.01 | 8.29 |
| 39.04° | (0 2 1) | 0.70 | 12.01 |
| 46.23° | (2 2 1) | 1.41 | 6.08 |
| 60.24° | (3 3 1) | 1.30 | 7.07 |
| 66.20° | (5 2 0) | 1.40 | 6.77 |
| Average crystallite sizes (nm) | 12.34 | | |

Supplementary Figure S1. N_2_ adsorption–desorption isotherms for the nano mullite.


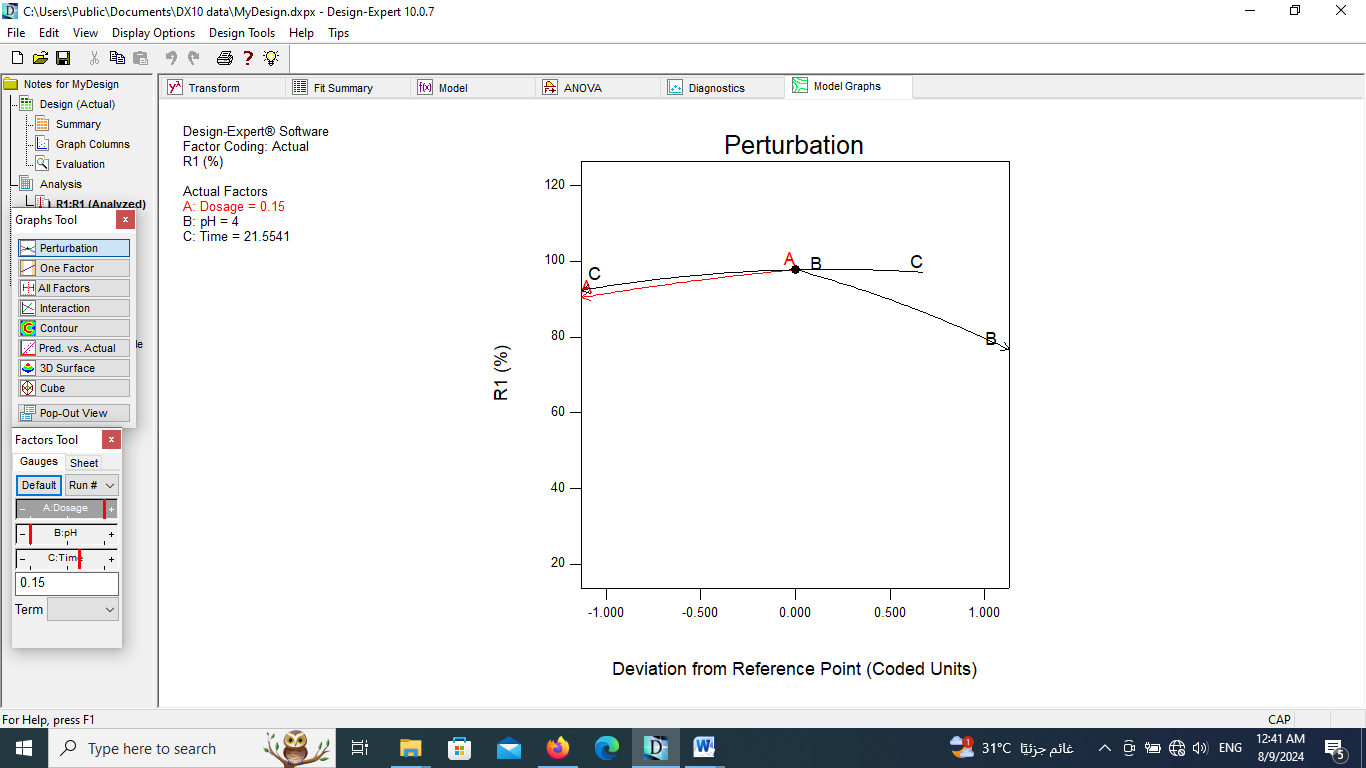


Supplementary Figure S2. The perturbation of the different parameters.
